# Bioinspired Design and Experimental Validation of an Aquatic Snake Robot

**DOI:** 10.3390/biomimetics9020087

**Published:** 2024-02-01

**Authors:** Giovanni Bianchi, Luca Lanzetti, Daniele Mariana, Simone Cinquemani

**Affiliations:** Dipartimento di Meccanica, Politecnico di Milano, Via La Masa 1, 20156 Milan, Italy; giovanni.bianchi@polimi.it (G.B.); luca.lanzetti@mail.polimi.it (L.L.); daniele.mariana@mail.polimi.it (D.M.)

**Keywords:** aquatic snake robot, anguilliform swimming, bioinspired design, underwater robotics, MPM-MLS algorithm

## Abstract

This article presents the design, simulation, and experimental validation of a novel modular aquatic snake robot capable of surface locomotion. The modular structure allows each unit to function independently, facilitating ease of maintenance and adaptability to diverse aquatic environments. Employing the material point method with the moving least squares (MPM-MLS) simulation technique, the robot’s dynamic behavior was analyzed, yielding reliable results. The control algorithm, integral to the robot’s autonomous navigation, was implemented to enable forward propulsion at high speed, steering, and obstacle detection and avoidance. Extensive testing of the aquatic snake robot was conducted, demonstrating its practical viability. The robot showcased promising swimming capabilities, achieving high speeds and maneuverability. Furthermore, the obstacle detection and avoidance mechanisms were proven effective, showing the robot’s ability to navigate through dynamic environments. The presented aquatic snake robot represents an advancement in the field of underwater robotics, offering a modular and versatile solution for tasks ranging from environmental monitoring to search and rescue operations.

## 1. Introduction

Locomotion in aquatic environments is particularly challenging for robots and autonomous vehicles, especially in unstructured spaces where obstacles and narrow passages are present [1,2]. Conversely, animals can move with extreme agility in unfamiliar and confined environments while maintaining good speed and energy efficiency [3,4]. Therefore, nature offers a valuable source of inspiration for the design of swimming robots, which is witnessing a growing interest among scientists and researchers [5,6,7,8,9,10].

Owing to their slender and flexible body, the swimming strategy adopted by aquatic snakes and eels is the most suitable in closed-quarter environments where turning rapidly and avoiding obstacles are requiredx [5]. Swimming robots inspired by these animals are extremely useful for operation in restricted locations where humans and larger remotely operated vehicles (ROVs) or autonomous underwater vehicles (AUVs) cannot access. A possible application is environmental monitoring since they can be equipped with sensors to collect data about temperature, water quality, or the presence of pollutants, and they can be equipped with cameras to observe marine life without disturbing animals. A different use of this robot involves inspecting underwater infrastructure such as pipelines, underwater cables, or even bridges and underwater platforms. Snake robots could also benefit the field of marine archaeology, being used to explore the seabed in search of artifacts or shipwrecks. Another important potential application of aquatic snake robots is search and rescue since the snake-like flexibility of the robot allows it to navigate through tight and confined spaces where traditional underwater vehicles might struggle. Finally, snake robots can be used for surveillance purposes, critical infrastructure protection, or border control [5,11].

Anguilliform locomotion is adopted by sea snakes, eels, and lampreys. It consists of a wave traveling from the animal’s head to its tail, pushing water backward and gaining thrust for momentum conservation, and the whole body undergoes large deformations and is involved in thrust generation [7,12,13]. An effective way to reproduce serpentine locomotion is to reproduce the snake’s body with a series of equal, rigid modules connected by joints, following the original idea of Hirose, who first developed a modular snake robot for terrestrial locomotion [14,15]. The first example of an underwater robot exploiting anguilliform locomotion is REEL-II, which is untethered and covered by a waterproof skin. It is composed of four rigid links connected by joints actuated by servomotors [16]. Similarly, Amphibot and its improved versions, Amphibot II and Amphibot III, are composed of several identical modules connected by cylindrical joints actuated by servomotors; these robots are amphibious since they can both swim in the water and crawl on the ground, and their control strategy is also inspired by snakes [11,17,18,19]. The same concept was applied in the development of the Salamandra Robotica II, which uses the same spine elements as the Amphibot for swimming; in addition, it has a passive flexible tail and four limbs [20]. Another amphibious robot is the Snake-like robot, which has two motors for each joint to perform complex swimming and crawling gaits [21]. Snakey is an aquatic modular robot designed for surface swimming, but its operational range is limited by its cable-based power and control system [22]. The SEA Snake is a modular snake robot with horizontal and vertical joints so that the robot is capable of 3D motion, and it contains series elastic actuators (SEAs) in each joint to allow compliant motion [23]. Mamba has a similar joint arrangement, and it is capable of sidewinding on the ground and swimming underwater; moreover, it has force and torque sensors embedded in the modules to improve its control [24]. Similar anguilliform-inspired robots use several modules and joints and an external cover for waterproofing [25,26]. Finally, AgnathaX is a modular snake robot actuated by servomotors and equipped with exteroceptive force sensors that measure the fluid dynamic forces and allow its bioinspired neural network to find the best swimming gait [27]. Although the majority of aquatic snake robots are modular and actuated by servomotors, some different designs can be adopted, such as a soft body actuated by shape memory alloy artificial muscles [28], fluidic elastomer actuators [29], or cables [6].

The robot described in this article is composed of eight modules actuated by servomotors, and each module is waterproof so that an external cover is not needed, making the external surface in contact with the water smooth and avoiding wrinkles that hinder the hydrodynamics. The shape of the robot’s head is unique, and it has been specifically designed to reduce the hydrodynamic drag. Furthermore, the robot is equipped with more powerful motors than the already existing robots, making it very robust even in challenging environments and allowing it to achieve high speeds. Moreover, another distinctive advantage of this robot is the presence of ultrasonic sensors in the head, which allow it to detect obstacles.

This article also describes a novel method of simulating robot dynamics and the complex fluid–structure interactions based on a moving least squares–material point method (MLS-MPM) algorithm implemented in Unity. This algorithm can solve the fluid dynamics in real time, allowing the simulation of many different swimming gaits and highlighting the effects of the kinematic parameters on swimming performance. Although the numerical accuracy is not comparable to that obtained with CFD simulation, the obtained results were used to find the parameters that allow for maximizing the swimming performances, which was the objective of this work.

The article is organized as follows: In Section 2, the kinematics of sea snake locomotion are described, and how it was reproduced with a modular robot is shown. In Section 3, the design of the robot is presented. In Section 4, the numerical analysis conducted to simulate its dynamics is shown. In Section 5, the control algorithm is described. In Section 6, the experimental results are presented. Finally, Section 7 is dedicated to the conclusions.

## 2. Kinematics of Anguilliform Swimming

Despite belonging to completely different classes, eels and aquatic snakes have similar shapes and swimming strategies, having long, slender bodies, involving all the body in thrust generation [3,7,12,13]. Anguilliform locomotion is a body–caudal fin (BCF) swimming gait, i.e., the animal’s movement consists of a wave propagating in a longitudinal direction from the head to the tail [3,12,13]. Since the cross-section is gradually variable along the longitudinal direction and no sharp edges are present, the flow remains attached to the body; thus, the surrounding water is pushed backward by the propagating wave, and the body gains thrust for momentum conservation [13].

The number of vertebrae in these animals is extremely high, ranging from 104 for eels to 186 for sea snakes [30], allowing them to generate serpentine curves with small radii and produce traveling waves with a short wavelength. Since the wavelengths are shorter than the body length, the motion is classified as undulatory [31].

The amplitude of the deformation *A* varies along the body length, and it can be modeled as follows [31]:

(1) $$A\left(\frac{x}{L}\right)=\alpha {\left(\frac{x}{L}\right)}^{2}+\beta \left(\frac{x}{L}\right)+\gamma$$

where *x* is the longitudinal coordinate, *L* is the body length, and α, β, and γ are coefficients that differ among animal species. In particular, sea snakes have an amplitude almost constant throughout the whole body length, whereas anguilliform fishes have an amplitude that increases significantly from the head to the tail. The deformation of the animal’s body in lateral direction *Z* can be expressed in the following form [31]:

(2) $$Z\left(\frac{x}{L},t\right)=A\left(\frac{x}{L}\right)\sin\left(\varphi \frac{x}{L}-\omega t\right)$$

where ω=2πf is the circular frequency, and ϕ is the wave number, which is defined as:

(3) $$\varphi =\frac{2\pi }{\lambda }$$

where λ is the wavelength.

The speed at which water is pushed backward is the one of the traveling wave, equal to λf. This means that the short wavelength λ, typical of anguilliform swimming, implies that the generated thrust is not as high as for different swimming strategies. Achieving a high wavelength could be possible but at the expense of a higher frictional drag on the body surface and higher axial forces increasing the stress on muscular tissues [31]. Although the speed achievable with anguilliform locomotion is slower than that of other strategies, there are several advantages. First, the lateral forces are always approximately balanced because having more than one period of the wave along the body implies that part of the body is pushing water laterally on one side while the other is on the opposite side. Moreover, the possibility of small-curvature radii allows rapid changes in direction, giving this swimming strategy the high maneuverability and agility needed to swim in narrow environments and in the presence of obstacles [6,32].

## 3. Design of the Robot

**Figure 1:**
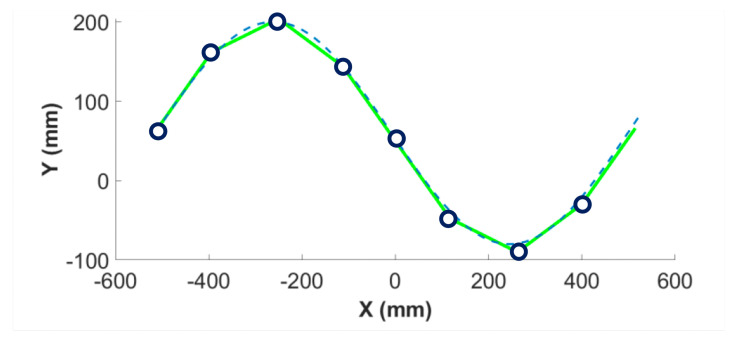
Approximation of the serpenoid curve with eight modules. The dashed line represents the serpenoid curve, the green solid lines represent the rigid modules, and the circles represent the joints between modules.

The objective of this work was to design an aquatic robot that can swim on the surface of water, mimicking anguilliform locomotion. To achieve this, the requirements listed below should be fulfilled:

- Modularity: The main requirement is the correct reconstruction of the serpentine curve, which is influenced by the number and the length of the modules of the robot. To obtain a snake robot that is 1.3 m long, it is possible to reproduce its shape with eight equally long modules connected by rotational joints and one module for the head. This way, the serpenoid curve for the typical amplitudes and wavelengths of these animals is accurately reproduced, as shown in Figure 1. Moreover, modularity implies that broken modules can be easily replaced and that other modules can be added to the robot without modifying the existing structure.
- Distributed actuation. Each module is independent and includes a power source and an actuator so that the malfunctioning of one module does not totally compromise the functioning of the robot.
- Buoyancy. The robot is supposed to move on the water surface in two dimensions. Thus, its buoyancy should be finely tuned to make the robot slightly buoyant so that it remains on the water surface, but the buoyancy should be minimal so that all the lateral surface of the robot is submerged, in contact with water, and can generate thrust. Moreover, the center of gravity of each module should be at the bottom to avoid rolling over.
- Absence of external cover. The choice of not using any external cover is driven by the fact that if this cover is not perfectly adherent to the robot, it folds during movement, creating wrinkles that hinder the hydrodynamic shape of the robot and letting the modules move inside the cover without displacing the surrounding water, also reducing the effectiveness of thrust generation. However, for the cover to adhere to the robot in any deformed shape, it must be elastic, and part of the torque given by the actuators is wasted on the cover deformation.
- Waterproofing. All the modules must be waterproof because they come into contact with water directly without any external cover, and any water leakage must be avoided to prevent damage to the electronic components.
- Remote control. The robot should be controlled remotely to eliminate the necessity for wires to communicate with the robot for control and to collect the data acquired by the sensors.

### 3.1. The Body Modules

The design of the modules is affected by the choice of not using any external cover, not only because of the waterproofing requirements but also because of the space between each module; the following must be minimized to prevent water from passing from one side to the other through this space and reducing the generated thrust. Thus, the modules have a buttonhole shape, the semicircumferences at their extremities allow for minimizing the space among the modules, and a clearance of only 1 mm is left.

**Figure 2:**
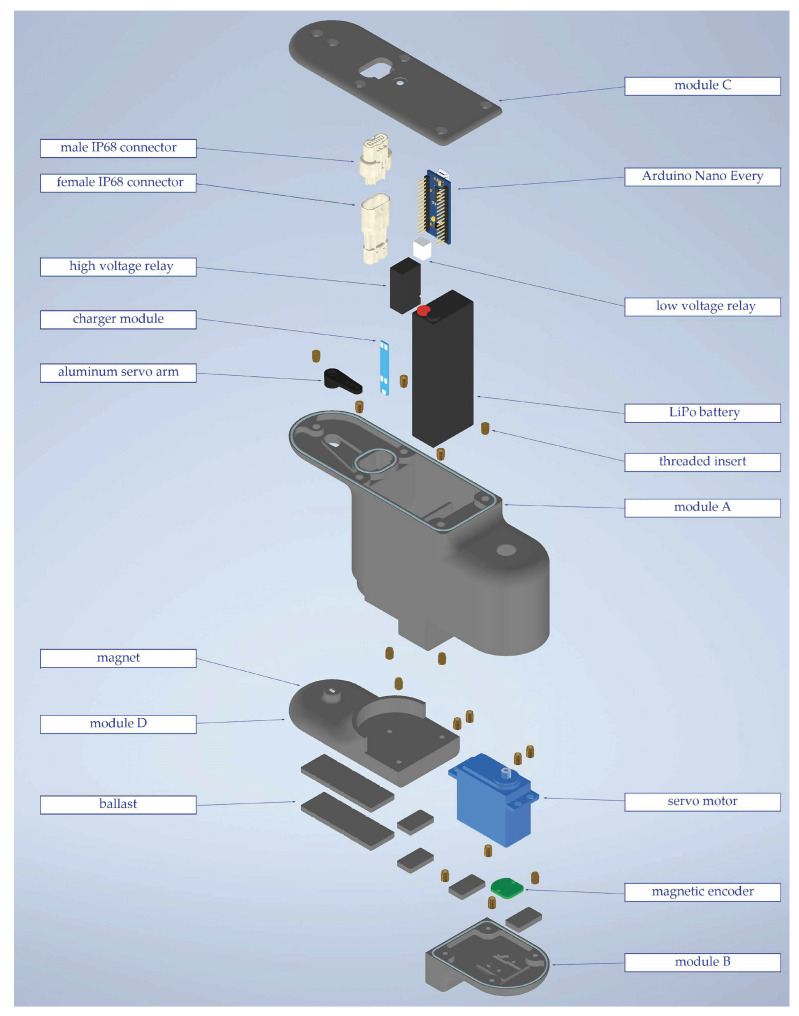
Exploded view of the module of the aquatic snake robot.

The modules were entirely 3D-printed and made of ABS. Each component, including connectors and servoarms, had a shape coupling with the module frame. The frame of the modules was composed of four parts (called A, B, C, and D in Figure 2), connected by screws using threaded inserts. An exploded view of the module with its components is shown in Figure 2.

Each module is independent of the others since it contains a power source, an electronic board, and an actuator. The components of each module are listed below:

- Servomotors. The selected servomotors were POWER-HD 40 waterproof, which could provide a torque of 3.96 Nm at 8.4 V and had a maximum speed of 60 rpm.
- Encoders. Since the chosen servomotors do not provide any position feedback, a magnetic encoder AS5600 was added. It was positioned on the motor axis at the bottom of each module, and a small cylindrical magnet was placed in the corresponding position of the previous module.
- Battery. The battery was a 2-cell LiPo, able to provide high current despite its small volume. In particular, the selected model was an OVONIC 2200 mAh 50 C.
- Battery charging circuit. The battery charging circuit included a BMS module that balances the two cells of the battery and an external circuit including diodes and relays, which allowed charging all the batteries of the robot by plugging in only one connector in the head, turning the robot on and off with only one switch in the head as well.
- Electronic board. The electronic board was an Arduino Nano Every, communicating through an I2C protocol with the robot’s head.

The waterproofing of the module’s external surface is achieved with a chemical treatment with acetone that fills the pores left by the 3D printing process. The connections between different parts of the module frame host an o-ring, and all the void space inside the module is filled with a dielectric gel. The buoyancy of the robot is adjusted by adding some iron ballasts at the bottom in a dedicated pocket. Each module’s mass is 0.6 kg, and it is 191 mm long, 60 mm wide, and 94 mm wide.

### 3.2. The Head Module

**Figure 3:**
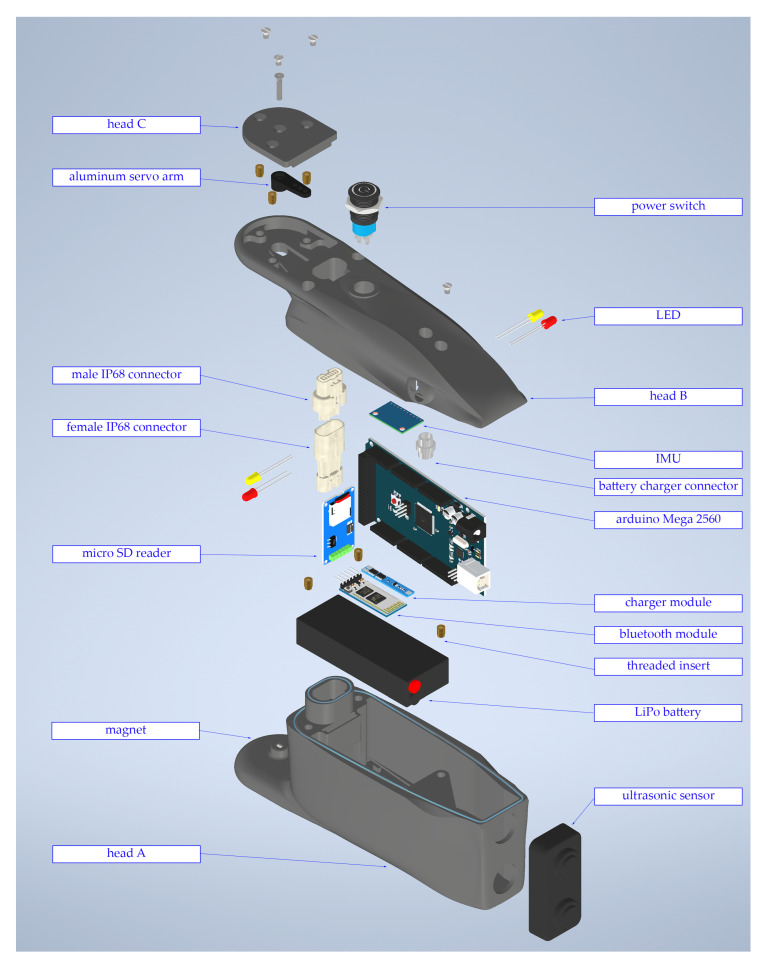
Exploded view of the head of the aquatic snake robot.

The head module featured some differences with respect to the body modules because the head hosted several sensors and was not equipped with a servomotor, so its geometry was slightly different. The external surface of the head was 3D-printed and made of ABS, and the waterproofing was ensured in the same way as for the modules. An exploded view of the head is shown in Figure 3.

The components present inside the head module are listed below:

- Battery and battery charging circuit. The battery and the BMS were the same as for the modules, but the recharging circuit was slightly different since the head hosted the connector for battery charging and the switch to turn on all the modules.
- Electronic board. The electronic board was an Arduino Mega, more powerful than the Arduino Nano present in the modules and able to manage more signals coming from all the sensors.
- Bluetooth module. The HC-05 Bluetooth module allowed the robot to communicate with the user, enabling them to give instructions and change the kinematic parameters while the robot is moving.
- IMU. The selected inertial measurement unit was the MP6050, which includes a triaxial accelerometer and a triaxial gyroscope.
- Ultrasonic sensor. An ultrasonic sensor was used to detect obstacles, and the chosen module was the HC-SR04, which is able to detect obstacles from a distance of 0.1 m to a distance of 2 m.
- SD card reader. The SD card was used to store the data collected from all the sensors since the large amount of collected data collected could not be transmitted in real time via serial communication through Bluetooth without undermining the control of the robot.

The head module’s mass was 0.65 kg, and it was 204 mm long, 60 mm wide, and 94 mm wide.

### 3.3. Robot Assembly

**Figure 4:**
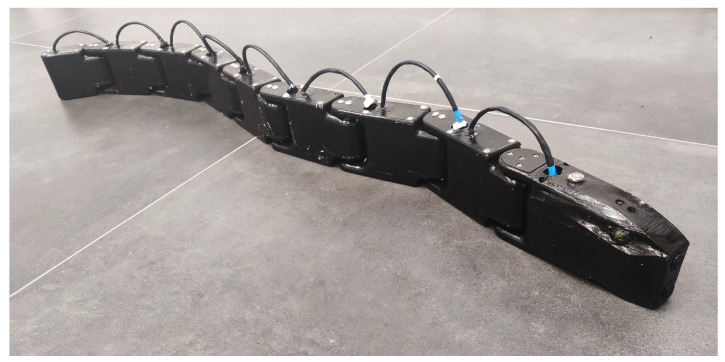
The assembled robot.

The assembled robot had an overall mass of 4.98 kg, and its total length was 1281 mm, while its width and height were 60 mm and 94 mm, respectively. The assembled robot is presented in Figure 4.

## 4. Numerical Analysis

The objective of this analysis was to understand the kinematic parameters, such as amplitude, frequency, and wavelength, that maximize the robot’s swimming performance. The level of complexity of the dynamics of the robot is remarkable since it is an articulated body composed of nine different rigid bodies interacting with a fluid. In addition, the number of motion laws’ parameter combinations is significant. Therefore, despite giving accurate results, a CFD approach is prohibitively time-consuming, so a different approach was chosen.

The simulation was created inside Unity, taking advantage of its built-in 3D physics engine, using C# programming language adopting the “MonoBehaviour.FixedUpdate()” function. The structure of the snake robot was created using the Articulation Body class, which can build hierarchically organized physics articulations and apply inverse dynamics. The moving least squares–material point method (MLS-MPM) algorithm used to simulate the water behavior was introduced to the simulation using the Zibra Liquids plug-in.

The MPM is a hybrid Lagrangian/Eulerian method in which the fluid is discretized as a huge number of particles, and a background grid is created over the whole domain. The particles’ mass and momentum are extrapolated to the grid points through shape functions, and the mass and momentum conservation are solved on the grid. Then, the velocity is extrapolated back to the particles, their derivatives are computed with the MLS approximation, and the forces exchanged by the fluid and the rigid bodies are computed. Finally, the velocity and the position of the particles and of the rigid bodies are updated, and the grid is reset [33,34].

The domain where the snake robot swims was a virtual tub of 3×1.5×0.25 m^3^ created with the Zibra Liquids plug-in. The grid resolution was set to 512, and about 5×10^6^ particles of 5 mm were simulated in real time with a fixed time step value Δt=0.02 s. The dimensions of the tub, the grid, and the particles were limited by the computational effort of simulating the fluid in real time. The articulation body was meshed with the Mephisto algorithm and scaled down using Blender. The inertial parameters of each component, the limit angles for each joint, and the maximum available torque for each motor were introduced to the simulation, and without a detailed servomotor characteristic curve, the torque–speed dependency was assumed to be linear. A liquid emitter and the articulation body were added to the container. In order to interact with the liquid, each object needed to have a rigid body component attached to it. However, this conflicted with the articulation body component already attached, so we needed to perform inverse dynamics. The method used by the plug-in code to add forces and torques to the rigid body component was the same as for the articulation body class: for this reason, the Zibra Liquid Collider code was accessed and modified in order to allow the application of force on each of the snake’s modules. Finally, the collider shape that interacted with the fluid particles was generated from the mesh of the snake robot using the Zibra Liquid server.

A first 60 s simulation was performed with the snake robot locked in the starting position and saved so that each simulation started from the same “Baked Liquid” configuration, where the fluid was at rest. The inputs to every simulation were the initial conditions, with the angular positions of each link as functions of time; the outputs were the torques of each joint and the displacement and rotation of the robot.

Two different C# codes were created: one to implement the eel-like movement, which is typical of anguilliform fishes, and the other for lateral undulation, typical of aquatic snakes. The user was able to set amplitude *A*, frequency *f*, and phase shift φ for each motion law, and the simulation’s duration was set to 10 s. The function “ArticulationBody.SetDriveTargets()”, calculates, for every time instant and for each drive, the angular position aim. For both motion laws, there was an initial exponential transient time to avoid discontinuities at the start of the simulation.

**Figure 5:**
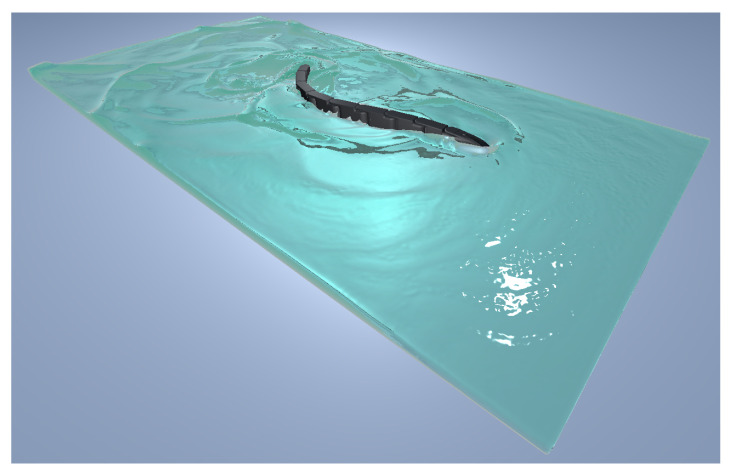
Snake robot performing eel-like motion.

The eel-like motion target angles for each drive were coded as:

(4) $$\theta_{i}=\left(1-e^{-\lambda k\Delta t}\right)A\frac{i-1}{N+1}\sin\left(\omega k\Delta t-i\varphi \right),$$

with i=1,…,8, and *N* being the total number of joints, equal to 8. This motion law is characterized by an amplitude that increases from the head to the tail, which is shown in Figure 5.

**Figure 6:**
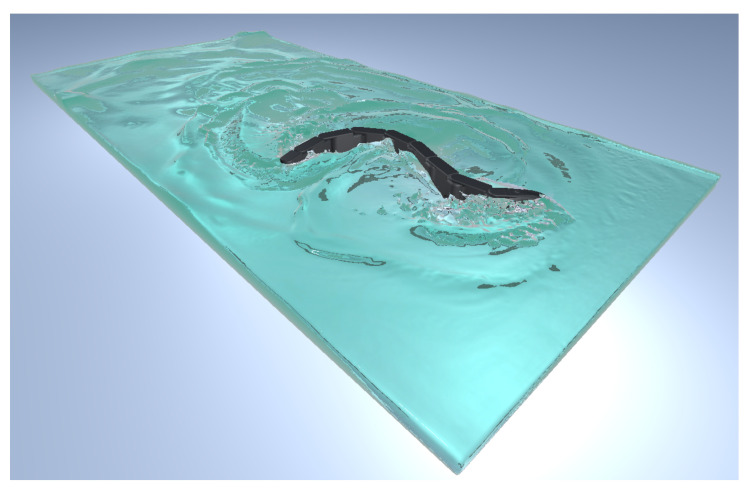
Snake robot performing lateral undulation motion.

Instead, the lateral undulation motion target angles for each drive were coded as follows, and the robot moving with this motion law is shown in Figure 6.

(5) $$\theta_{i}=\left(1-e^{-\lambda k\Delta t}\right)A\sin\left(\omega k\Delta t-i\varphi \right),$$

with i=1,…,8.

The simulation results presented in this article refer to lateral undulation motion law since the effects of the kinematic parameters are very similar, but lateral undulation better highlights the phenomena involved. The range of parameters was chosen according to the data shown in Table 1 for a total of 1764 simulations.

### Simulation Results

**Figure 7:**
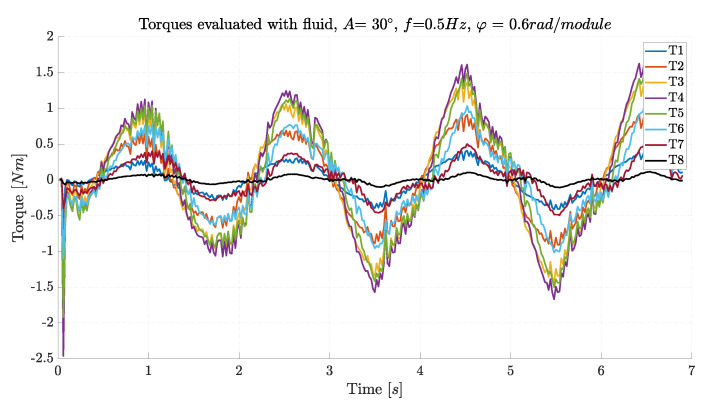
Motor torques for lateral undulation.

The inverse dynamics calculation was implemented in Unity using the command “ArticulationBody.GetDriveTorques()”, and the torques applied to each joint are presented in Figure 7.

The motor torques are sinusoidal following the motion law, and even though the amplitude of rotation is equal, the required torque is the minimum for the first and last joints and a maximum in the center of the robot.

**Figure 8:**
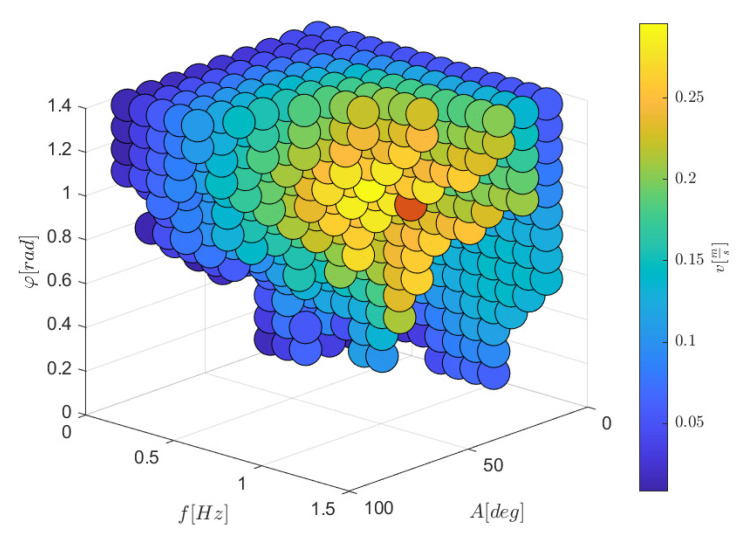
Snake robot velocity for different kinematic parameters. The combination of parameters giving the highest velocity is highlighted in red.

The steady-state value of the robot speed for each simulation is shown in Figure 8. The combinations missing in the plot were characterized by torque saturation or self-collisions of the robot.

A speed trend emerged, highlighting an optimal yellow zone with a red dot representing the maximum achievable speed of 0.30 m/s.

Increasing the amplitude *A* has a beneficial effect until the motors saturate since more fluid is displaced, increasing the forces generated.

An increase in the frequency *f* has a twofold effect on the achieved speed: the higher the frequency, the higher the speed of the traveling wave that accelerates backward the surrounding water, so more thrust is generated; however, the inertia forces of the robot increase as $\omega^{2}$, leading to saturation of the motors and making the motion law unfeasible at high frequencies.

A high phase shift entails a short wavelength and a low traveling wave speed, so increasing the phase shift reduces the generated thrust. However, this is valid only when the phase shift remains above ∼0.6 rad/module. When it decreases under this threshold, the fluid forces are more directed laterally than longitudinally: not only is a significant part of the power wasted, but the lateral displacement of the robot and its rotation also makes the robot movement different from the desired one. Furthermore, a small phase shift φ among modules means that all the joints have similar angles, and the robot tends to self-collide even for small amplitudes. Therefore, the highest swimming velocity is achieved when the robot has a phase shift of 0.9 rad/module, and the amplitude and the frequency are the maximum possible without saturating the motors, i.e., $30^{\circ }$ and 0.9 Hz.

## 5. Control Algorithm

**Figure 9:**
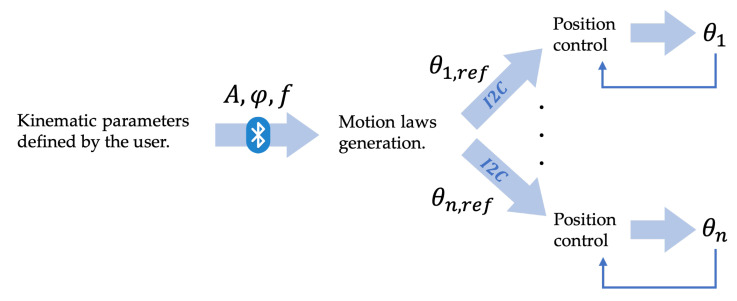
Signal transfer route.

The control of the robot’s movement performs two tasks: low-level feedback motor control and motion law generation, as shown in Figure 9.

### 5.1. Motor Control

The motor control was carried out by the Arduino Nano boards in each module, ensuring that the motors reached the desired angle. Thanks to their built-in PID controllers, the adopted servomotors exhibited high precision in reaching target angles. However, in order to eliminate reliance on unknown components, an encoder was used to provide real-time angular position measurement, which could be fundamental for developing more complex control strategies in future work.

### 5.2. Motion Law Generation

The motion law generation was performed by the Arduino Mega in the robot head, following the instructions received by the user via Bluetooth. The angles required for each joint were evaluated according to Equations (4) and (5) and communicated through the I2C protocol to the modules. To steer the robot, a phase offset equal to all the modules was added to Equations (4) and (5).

(6) $$\theta_{i}=\left(1-e^{-\lambda t}\right)A\sin\left(i\varphi -\omega t\right)+\varphi_{offset},$$

**Figure 10:**
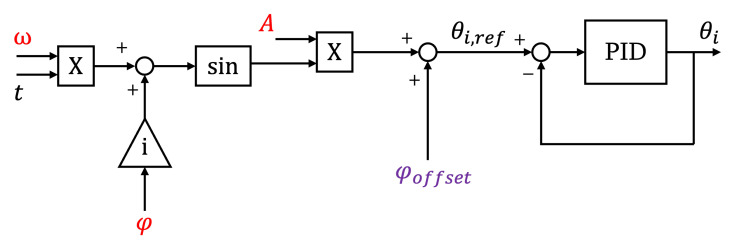
Block diagram of the control algorithm.

In Figure 10, the block diagram of the control algorithm for the *i*thmodule is presented. The inputs provided by the user are shown in red, and the offset angle $\varphi_{offset}$, highlighted in violet, could be either provided by the user for steering or computed by the robot’s head if obstacle avoidance was activated, as shown in the following subsection.

### 5.3. Obstacle Avoidance

The position of obstacles is not known in advance, and the robot position is not measured; thus, the strategy for obstacle avoidance cannot be based on trajectory planning. However, thanks to the feedback signal of the ultrasonic sensor, the robot can recognize when an obstacle is present on its current trajectory and steer to avoid collisions.

To correctly overcome obstacles, two fundamental pieces of information are needed: the measurement of the obstacle distance from the robot’s head, measured with an ultrasonic sensor, and the obstacle position with respect to the current trajectory. The latter is obtained by measuring the angular position of the first joint in the instant the obstacle is detected. The head of the robot, especially in lateral undulation, has a small periodical rotation even during forward swimming, so despite the ultrasonic sensor pointing only straight ahead, the obstacles can be detected in a wide angular range corresponding to ±A. The position of the obstacle is used to decide whether to turn left or right, whereas the distance from the obstacle is used to compute how sharply it is necessary to turn. The curvature radius is influenced by a phase offset angle called $\varphi_{obstacle}$, which is calculated as follows:

(7) $$\theta_{i}=A\sin\left(i\varphi -\omega t\right)+\varphi_{obstacle}$$

where $\varphi_{obstacle}$ is

(8) $$\varphi_{obstacle}=-\left(\frac{\theta_{encoder}}{|\theta_{encoder}|}\right)\frac{d}{D}K$$

where $\theta_{encoder}$ is the angle measured by the encoder placed in the head of the robot when the obstacle is detected, $\frac{d}{D}$ is the ratio between the distance of the obstacle detected by the sensor and the maximum distance that can be measured, and *K* is a gain that increases the effect of control action. It is important to notice that summing $\varphi_{obstacle}$ at every time instant is not possible because continuous variations in offset severely affect the original reference signal, leading to completely inefficient motion. For this reason, after the evaluation of the shift needed, $\varphi_{obstacle}$ is summed to the reference signal as a constant value for a fixed amount of time *T*, and the optimal value of *T* is found experimentally to have the best combination of promptness and smoothness of motion. Furthermore, another exponential transient is added to erase any sudden variation completely, and $\varphi_{obstacle}$ is evaluated according to Equation (9).

(9) $$\left\{\begin{array}{c} \varphi_{obstacle}\left(t\right)=-\left(1-e^{-\lambda t}\right)\left(\frac{\theta_{encoder}}{|\theta_{encoder}|}\right)\frac{d}{D}K),\;t\in \left[0,\frac{T}{2}\right)\\ \varphi_{obstacle}\left(t\right)=-e^{-\lambda (t-\frac{T}{2})}\left(\frac{\theta_{encoder}}{|\theta_{encoder}|}\right)\frac{d}{D}K),\;t\in \left[\frac{T}{2},T\right) \end{array}\right.$$

**Figure 11:**
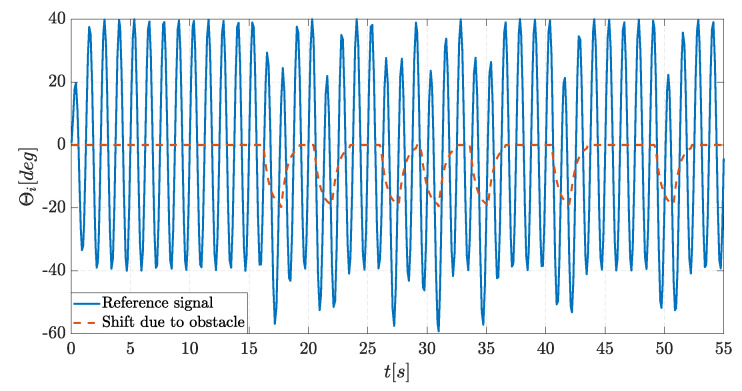
Referencesignal for lateral undulation while avoiding obstacles.

The reference angle computed in the presence of an obstacle is shown in Figure 11, where the obstacle is positioned on the left of the trajectory, T=3 s, and K=20.

## 6. Experimental Results

The experiments were aimed at verifying the ability of the robot to perform the following tasks:

- Forward swimming;
- Steering;
- Obstacle avoidance.

Only the results obtained with lateral undulation are shown to avoid redundancy of data. The results obtained with eel-like motion were analogous; the only significant difference was that to obtain a speed similar to lateral undulation, it was necessary to have a higher amplitude and frequency.

### 6.1. Forward Swimming

**Figure 12:**
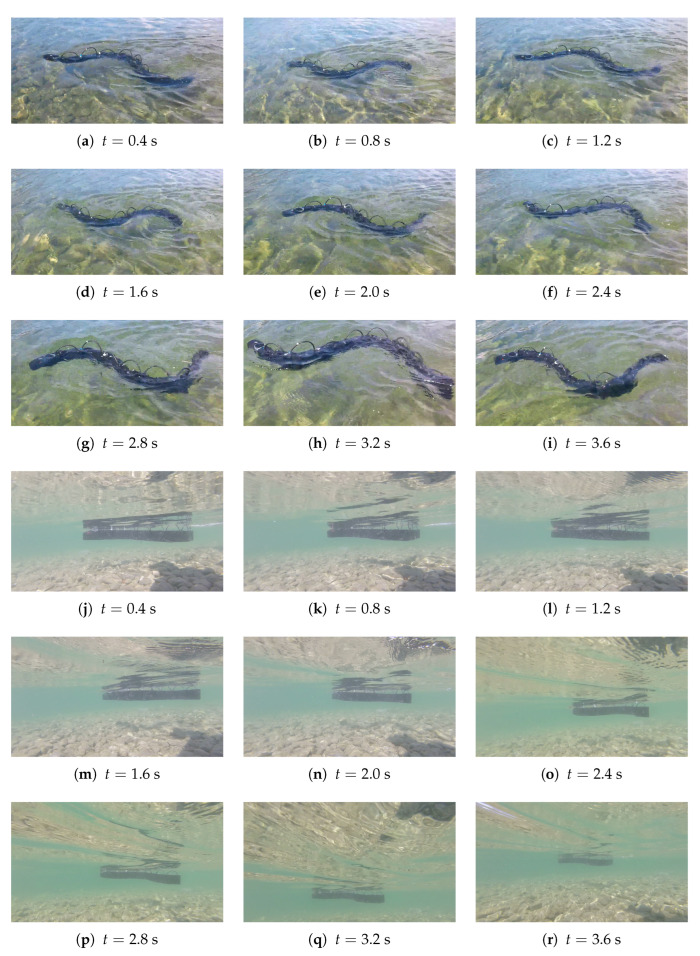
Experimental test for maximum speed obtained from the two different perspectives: each time of the frame is specified under the image, f=0.9 Hz, A=30°, φ=0.9 rad.

The robot was capable of swimming, and it reached a speed of 0.39 m/s for a frequency of 0.9 Hz, an amplitude of $30^{\circ }$, and a phase shift of 0.9 rad, as shown in Figure 12. The velocity obtained was in accordance with the simulation results, meaning that despite the simplifying assumptions, the numerical model was capable of correctly simulating the principal aspects of the problem. For these tests, the average velocity was calculated by attaching the head of the robot to a wire that unwound as the robot proceeded forward and measuring the traveled distance and the elapsed time. All these tests had a duration of >40 s to minimize the effect of the initial transient on the average speed calculation.

The robot was tested with different kinematic parameters, and the average speed for each test is shown in Table 2 and compared to the result obtained from the simulation. It can be observed that the simulation slightly underestimated the speed reached by the robot, but there is a substantial similarity between the experimental and the numerical results.

### 6.2. Steering

**Figure 13:**
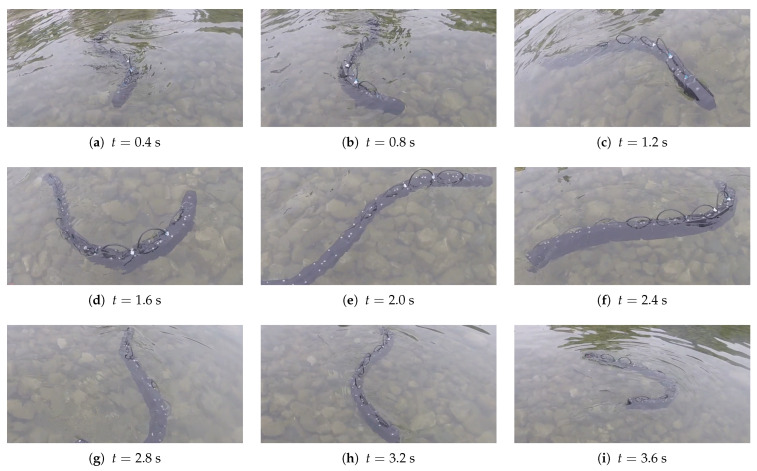
Experimental test for steering with constant offset method; the time of each frame is specified under the image, f=0.5 Hz, A=20°, φ=π/6 rad.

The experiments confirmed that the constant offset strategy effectively performed turns, as shown in Figure 13.

### 6.3. Obstacle Avoidance

**Figure 14:**
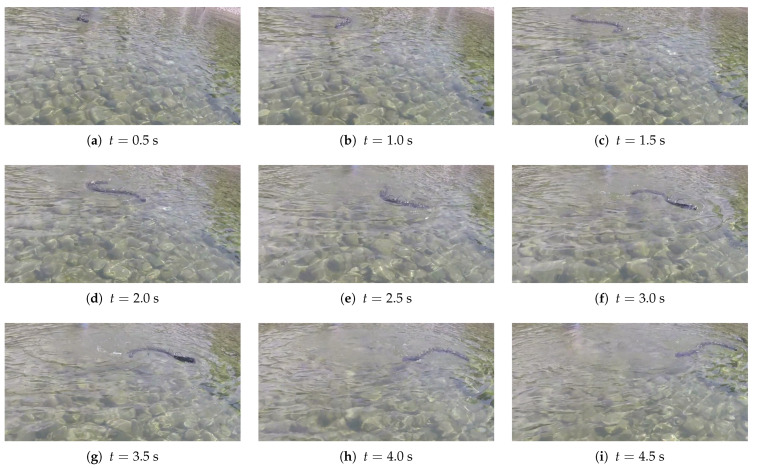
Experimental test for obstacle avoidance; the time of each frame is specified under the image, f=0.5 Hz, A=20°, φ=π/6 rad.

Finally, the ability of the robot to avoid obstacles was assessed, verifying the efficacy of the designed control algorithm. In Figure 14, it can be observed how the robot detected an obstacle represented by the camera operator from a distance of 1.5 m and turned to avoid it.

## 7. Conclusions

The developed snake robot can reach a high speed and avoid obstacles with the feedback control realized using an ultrasonic sensor. This promising swimming performance was obtained owing to the low inertia of the modules and the powerful motors. The robot is practical from the user’s perspective and is completely waterproof since no damage or practical issues emerged from the presence of water during the experiments.

While acknowledging certain similarities with the already existing snake robots, it is possible to highlight the unique contributions and enhancements provided by the presented design. The robot’s actuation mechanism shares a modular design that is common in many snake robots. However, a notable enhancement lies in integrating waterproof and more powerful motors. Compared with Amphibot III [19] and Mamba [24], this robot’s actuation system allows for greater force generation, resulting in robust locomotion, especially in challenging aquatic environments. Using an ultrasonic sensor to detect obstacles is common in underwater robotics, but it was not included in the previously developed snake robots. The obstacle avoidance algorithm employed establishes fundamental capabilities despite being extremely simple, and future iterations of this work may explore more sophisticated algorithms to enhance the robot’s adaptability. Thus, although our aquatic snake robot shares common features with existing designs, the integration of more powerful motors and the presence of sensors for obstacle detection constitutes a novelty in aquatic robotics with potential applications in diverse environments. Moreover, the developed simulation effectively handles the interaction between the fluid and the robot’s structure and is significantly faster than other strategies. The speed and torque values derived from this simulation were tested and demonstrated consistency when evaluated within the context of the underlying hypotheses. In future work, the robot will be equipped with GPS sensors to effectively control its trajectory, allowing the development of more advanced control algorithms that calculate kinematic parameters (amplitude, frequency, and phase shift) autonomously according to the desired speed and trajectory.

## Figures and Tables

**Table 1:** List of parameters for simulation tests.

	Minimum	Maximum	Unit
***A***	10	90	deg
***f***	0.1	1.4	Hz
**φ**	0.1	1.4	rad

**Table 2:** Comparison of numerical and experimental results.

*A*	*f*	φ	$v_{\exp}$	$v_{\mathrm{num}}$
$20^{\circ }$	0.7 Hz	0.5 rad	0.18–0.24 m/s	0.23 m/s
$20^{\circ }$	0.9 Hz	0.9 rad	0.24–0.27 m/s	0.22 m/s
$30^{\circ }$	0.9 Hz	0.9 rad	0.35–0.42 m/s	0.30 m/s
$40^{\circ }$	0.9 Hz	0.9 rad	0.32–0.39 m/s	0.25 m/s
$50^{\circ }$	0.9 Hz	0.9 rad	0.30–0.32 m/s	0.23 m/s
$60^{\circ }$	0.9 Hz	0.9 rad	0.24–0.27 m/s	-
$60^{\circ }$	1.3 Hz	0.9 rad	0.37–0.44 m/s	-

## Data Availability

No new data were created or analyzed in this study. Data sharing is not applicable to this article.

## References

[1] Kelasidi E., Pettersen K., Gravdahl J., Stromsoyen S., Sorensen A. Modeling and Propulsion Methods of Underwater Snake Robots. Proceedings of the 2017 IEEE Conference on Control Technology and Applications (CCTA).

[2] Sverdrup-Thygeson J., Kelasidi E., Petersen K., Gravdahl J. A control framework for biologically inspired underwater swimming manipulators equipped with thrusters. Proceedings of the 10th IFAC Conference on Control Applications in Marine Systems CAMS 2016.

[3] Lighthill M. (1969). Hydromechanics of Aquatic Animal Propulsion. Annu. Rev. Fluid Mech..

[4] Triantafyllou M., Triantafyllou G. (1995). An Efficient Swimming Machine. Sci. Am..

[5] Kelasidi E., Liljeback P., Pettersen K., Gravdahl J. (2016). Innovation in underwater robots: Biologically Inspired Swimming Snake Robots. IEEE Robot. Autom. Mag..

[6] Gautreau E., Bonnet X., Sandoval J., Fosseries G., Herrel A., Arsicault M., Zeghloul S., Laribi M. (2022). A biomimetic Method to Replicate the Natural Fluid Movements of Swimming Snakes to Design Aquatic Robots. Biomimetics.

[7] Salazar R., Fuentes V., Abdelkefi A. (2018). Classification of biological and bioinspired aquatic systems: A review. Ocean Eng..

[8] Wang Y., Chen H., Law J., Du X., Yu J. (2023). Ultrafast Miniature Robotic Swimmers with Up-Stream Motility. Cyborg Bionic Syst..

[9] Chuang C., Zhang Y., Wang W., Xi N., Liu L. (2022). A Manta Ray-Inspired Biosyncretic Robot with Stable Controllability by Dynamic Electric Stimulation. Cyborg Bionic Syst..

[10] Cao Q., Wang R., Zhang T., Wang Y., Wang S. (2022). Hydrodynamic Modeling and Parameter Identification of a Bionic Underwater Vehicle: RobDact. Cyborg Bionic Syst..

[11] Crespi A., Badertscher A., Guignard A., Ijspeert A. Swimming and Crawling with an Amphibious Snake Robot. Proceedings of the 2005 IEEE International Conference on Robotics and Automation.

[12] Sfakiotakis M., Lane D., Davies J. (1999). Review of Fish Swimming Modes for Aquatic Locomotion. IEEE J. Ocean. Eng..

[13] Wu T. (1971). Hydromechanics of Swimming of Fishes and Cetaceans. Adv. Appl. Mech..

[14] Hirose S. (1993). Biologically Inspired Robots: Snake-Like Locomotors and Manipulators.

[15] Hirose S., Mori M. Biologically Inspired Snake-like Robots. Proceedings of the 2004 IEEE International Conference on Robotics and Biomimetics.

[16] McIsaac K., Ostrowski J. A Geometric Approach to Anguilliform Locomotion: Modelling of an Underwater Eel Robot. Proceedings of the 1999 IEEE International Conference on Robotics & Automation.

[17] Crespi A., Badertscher A., Guignard A., Ijspeert A. An amphibious robot capable of snake and lamprey-like locomotion. Proceedings of the 35th International Symposium on Robotics (ISR 2004).

[18] Crespi A., Ijspeert A. Amphibot II: An Amphibious Snake Robot that Crawls and Swims Using a Central Pattern Generator. Proceedings of the 9th International Conference on Climbing and Walking Robots.

[19] Porez M., Boyer F., Ijspeert A. (2014). Improved Lighthill fish swimming model for bio-inspired robots—Modelling, computational aspects and experimental comparisons. Int. J. Robot. Res..

[20] Crespi A., Karakasiliotis K., Guignard A., Ijspeert A. (2013). Salamandra Robotica II: An Amphibious robot to Study Salamander-like Swimming and Walking Gaits. IEEE Trans. Robot..

[21] Yu S., Ma S., Li B., Wang Y. An Amphibious Snake-like Robot: Design and Motion Experiments on Ground and in Water. Proceedings of the 2009 IEEE International Conference on Information and Automation.

[22] Jasni M., Samin R., Ibrahim B. (2012). Biological Inspired Inspection Underwater Robot (SNAKEY). Procedia Eng..

[23] Rollinson D., Bilgen Y., Brown B., Enner F., Ford S., Layton C., Rembisz J., Schwerin M., Willig A., Velagapudi P. Design and Architecture of a Series Elastic Snake Robot. Proceedings of the 2014 IEEE/RSJ International Conference on Intelligent Robots and Systems.

[24] Liljeback P., Stavdahl O., Pettersen K., Gravdahl J. Mamba—A Waterproof Snake Robot with Tactile Sensing. Proceedings of the 2014 IEEE/RSJ International Conference on Intelligent Robots and Systems.

[25] Raj A., Kumar A., Thakur A. Automated Locomotion Parameter Tuning for an Anguilliform-inspired Robot. Proceedings of the 2016 IEEE International Conference on Systems, Man, and Cybernetics.

[26] Niu X., Xu J., Ren Q., Wang Q. (2013). Locomotion Generation and Motion Library Design for an Anguilliform Robotic Fish. J. Bionic Eng..

[27] Thandiackal R., Melo K., Paez L., Herault J., Kano T., Akiyama K., Boyer F., Ryczko D., Ishiguro A., Ijspeert A. (2021). Emergence of robust self-organized undulatory swimming based on local hydrodynamic force sensing. Sci. Robot..

[28] Ayers J., Wilburs C., Olcott C. Lamprey Robots. Proceedings of the International Symposium on Aquabiomechanisms.

[29] Feng H., Sun Y., Todd P., Lee H. (2019). Body Wave Generation for Anguilliform Locomotion Using a Fiber-Reinforced Soft Fluidic Elastomer Actuator Array toward the Development of the Eel-Inspired Underwater Soft Robot. Soft Robot..

[30] Gillis G. (1996). Undulatory Locomotion in Elongate Aquatic Vertebrates: Anguilliform Swimming since Sir Gray. Am. Zool..

[31] Khalid M., Wang J., Akhtar I., Dong H., Liu M., Hemmati A. (2021). Why do anguilliform swimmers perform undulation with wavelengths shorter than their bodylengths?. Phys. Fluids.

[32] Xu J., Niu X., Ren Q. (2012). Modeling and Control Design of an Anguilliform robotic Fish. Int. J. Model. Simul. Sci. Comput..

[33] Jiang C., Schroeder C., Teran J., Stomakhin A., Selle A. The material point method for simulating continuum materials. Proceedings of the ACM SIGGRAPH 2016 Courses.

[34] Hu Y., Fang Y., Ge Z., Qu Z., Zhu Y., Pradhana A., Jiang C. (2018). A moving least squares material point method with displacement discontinuity and two-way rigid body coupling. ACM Trans. Graph..

